# Association between monocyte/high-density lipoprotein cholesterol ratio and carotid plaque in postmenopausal women: A cross-sectional study

**DOI:** 10.1097/MD.0000000000037425

**Published:** 2024-03-22

**Authors:** Jing Guo, Haibo Qin, Xianxian Li

**Affiliations:** a Health Management Center, Heping Hospital affiliated to Changzhi Medical College, Changzhi, Shanxi, China; b Department of Cardiovascular Medicine, Heping Hospital affiliated to Changzhi Medical College, Changzhi, Shanxi, China; c Department of Hematology, Heji Hospital affiliated to Changzhi Medical College, Changzhi, Shanxi, China.

**Keywords:** carotid atherosclerosis, inflammation, monocyte/HDL cholesterol, plaque, postmenopausal women

## Abstract

To investigate the relationship between carotid plaque and monocyte to high-density lipoprotein cholesterol ratio (MHR) in postmenopausal women. A cross-sectional study was conducted and 214 postmenopausal women who underwent physical examination at the Health Management Center of Heping Hospital affiliated to Changzhi Medical College between August 2018 and August 2022 were enrolled. The subjects were grouped according to the results of carotid ultrasound. The general information, blood pressure, biochemical markers, and routine blood indicators were compared between the 2 groups. Binary logistic regression was performed to analyze the correlation between MHR and carotid plaque in postmenopausal women, and receiver operating characteristics (ROC) curve was used to analyze the predictive value of MHR for carotid plaque in this population. The carotid plaque group showed a lower high-density lipoprotein cholesterol (HDL-C) (1.21 [1.08–1.425] vs 1.29 [1.15–1.445] mmol/L, Z = −2.115, *P* = .034) and a higher MHR [0.33 ± 0.1 vs 0.26 ± 0.1, t = −5.756, *P* < .001] when compared to the no carotid plaque group. After adjusting for potential confounders such height, weight, and HDL-C, binary logistic regression analysis revealed that MHR continued to be an independent risk factor for the formation of carotid plaque in postmenopausal women (odds ratio [OR] = 1.795, 95% confidence interval [CI] 1.198–2.689, *P* = .005). ROC curve analysis indicated that MHR had a 95% CI of 0.656 to 0.793 in predicting carotid plaque formation, an optimal cut-point of 0.265, and a sensitivity and specificity of 82.2% and 58.9%, respectively. MHR is a distinct risk factor for carotid plaque formation in postmenopausal women.

## 1. Introduction

Atherosclerosis (AS) underlies the pathological basis of cardiovascular and cerebrovascular events, representing a serious health crisis worldwide. Carotid ultrasound is now available for early detection of AS and contributes to timely clinical intervention.^[1]^ Carotid ultrasound as an effective and noninvasive evaluation method of carotid AS, is often considered an entry point to reflect systemic atherosclerotic lesions.^[2]^ Through in-depth exploration on the etiology and pathogenesis of AS, it is generally accepted that AS is essentially an inflammatory lesion. The monocytes to high-density lipoprotein cholesterol ratio (MHR) has been identified as a novel inflammatory marker combining the 2 characteristics.^[3,4]^ Currently, the majority of studies on MHR and AS gives priority to the general population, with few studies focusing on postmenopausal women. Herein, the present study aims to investigate the association between MHR and carotid plaque in postmenopausal women and to explore the value of MHR for predicting AS in this population.

## 2. Materials and methods

### 2.1. Study subjects

This cross-sectional study recruited 214 postmenopausal women aged 52 to 88 years old, with an average age of 61.62 ± 8.68, who underwent physical examination at the Health Management Center of Heping Hospital affiliated to Changzhi Medical College from August 2018 to August 2022. These subjects were assigned to the no carotid plaque group (107 cases) and the carotid plaque group (107 cases) according to their carotid vascular ultrasound images. Inclusion criteria: postmenopausal female population; complete general examination, laboratory examination, and carotid vascular ultrasound data. Exclusion criteria: lipid-regulated drug treatment; non-atherosclerotic lesions such as arteritis; presence of acute signs of infection; combination of liver, kidney, brain diseases, malignant tumors, haematological and autoimmune diseases, acute, and chronic infectious diseases. The Ethics Committee of Heping Hospital affiliated to Changzhi Medical College gave its approval to the project (approval number: 2023-021).

### 2.2. Methods

#### 2.2.1. General data collection.

The basic information of enrolled subjects such as medical history, age, and blood pressure at the time of physical examination, waist circumference, hip circumference, height, and weight were recorded, and the body mass index (BMI) was calculated: BMI = weight (kg)/height (m^2^). The blood pressure was measured in strict accordance with the standards of the Chinese Guidelines for the Prevention and Treatment of Hypertension (2018 Revision).^[5]^ All enrolled patients were allowed at least 5 minutes of quiet rest before blood pressure measurement at admission, and the blood pressure was measured every 5 minutes in a seated upper arm position (upper arm at heart level) for a total of 3 times. Three measurements of blood pressure were recorded by the same experienced nurse using an Omron electronic sphygmomanometer, including systolic and diastolic blood pressure.

#### 2.2.2. Laboratory tests.

After 12 hours of fasting, 5 mL of peripheral venous blood was drawn from the patients in the morning for examination. Laboratory indicators such as leukocytes, neutrophils, monocytes, serum high-density lipoprotein cholesterol (HDL-C), serum low-density lipoprotein cholesterol, blood glucose, and serum creatinine were tested and recorded using a fully automated biochemical instrument in the laboratory department of our hospital, and the MHR was calculated.

#### 2.2.3. Carotid color Doppler ultrasound examination.

ML6-15 superficial line array probe of a professional color Doppler ultrasound diagnostic instrument (GE VIVID E90, USA) with a probe frequency of 6 to15 MHz was used to scan the carotid bifurcation, internal carotid artery, external carotid artery, and common carotid artery of patients in a supine position. The intima-media thickness (IMT), size, number, and location of plaques were recorded. Plaque was defined as a focal structure that encroaches into the arterial lumen of at least 0.5 mm or 50% of the surrounding IMT value or demonstrates a thickness of >1.5 mm as measured from the media-adventitia interface to the intima-lumen interface.^[6]^

### 2.3. Statistical methods

All data were statistically processed using SPSS 26.0 software. Kolmogorov-Smirnov test was used for evaluating the normality of continuous variables. Data conforming to the normal distribution were expressed as mean ± standard deviations (*x* ± s), and inter-group comparisons were performed using the independent sample *t* test. Data that did not meet the normal distribution were expressed as median (*P*_25_–*P*_75_), and inter-group comparisons were made using the nonparametric test and Mann–Whitney *U* rank sum test for 2 independent samples. The categorical variables were presented as frequencies and percentages (%) and intergroup comparisons were made using the Chi-Square test or Fisher exact test. Carotid plaque risk factors in postmenopausal women were analyzed using the method of binary logistic regression; the optimal threshold value of MHR in postmenopausal women was predicted by the receiver operating characteristic (ROC) curve. A value of *P* < .05 was considered statistically significant.

## 3. Results

### 3.1. Clinical profiles of postmenopausal women in the no carotid plaque group versus the carotid plaque group

Compared to those in the no carotid plaque group, patients in the carotid plaque group had higher levels of age, BMI, waist-to-hip ratio, leukocytes, neutrophils, lymphocytes, monocytes, and triglycerides, and lower levels of HDL-C (all *P* < .05), but there was no statistical difference in the comparison of remaining indicators, as shown in Table 1.

**Table 1:** Clinical information of no carotid plaque group and the carotid plaque group in postmenopausal women.

Project	Carotid plaque-free group (107 cases)	Carotid plaque group (107 cases)	Z/*t*/X^2^ values	*P*-value
Age (yr)	55 (53, 58)	67 (59.5, 72)	−9.144	<.001
BMI (kg/m^2^)	23.81 ± 3.04	25.03 ± 3.15	−2.901	.004
Waist-to-hip ratio	0.85 ± 0.06	0.89 ± 0.07	−5.026	<.001
With hypertension	96 (89.72)	55 (51.4)	42.414	<.001
No hypertension	11 (10.28)	52 (48.6)		
With diabetes	104 (97.2)	90 (84.11)	10.81	.001
No diabetes	3 (2.8)	17 (15.89)		
With fatty liver	69 (64.49)	56 (52.34)	2.984	.084
No fatty liver	38 (35.51)	50 (46.73)		
Leukocytes (×10^9^/L)	5.1 (4.5, 6.2)	6.1 (5.3, 7.25)	−4.626	<.001
Neutrophils (×10^9^/L)	2.98 (2.3, 3.685)	3.6 (2.855, 4.365)	−4.031	<.001
Lymphocytes (×10^9^/L)	1.82 (1.505, 2.02)	2.02 (1.67, 2.425)	−3.448	.001
Monocytes (×10^9^/L)	0.31 (0.25, 0.37)	0.4 (0.34, 0.47)	−5.779	<.001
TG (mmol/L)	1.3 (1.095, 1.84)	1.7 (1.3, 2.135)	−3.144	.002
TC (mmol/L)	5.41 ± 0.71	5.54 ± 0.92	−1.185	.237
LDL-C (mmol/L)	3.4 (3.085, 3.83)	3.52 (3.105, 4.005)	−1.202	.229
HDL-C (mmol/L)	1.29 (1.15, 1.445)	1.21 (1.08, 1.425)	−2.115	.034
Creatinine (µmol/L)	60 (55, 65)	59 (52, 65)	−1.4	.161
Uric acid (µmol/L)	290 (253, 331.5)	281 (241, 355.5)	−0.15	.881
Blood glucose (mmol/L)	5.32 (4.96, 5.74)	5.42 (5.025, 6.01)	−1.668	.095
MHR	0.26 ± 0.1	0.33 ± 0.1	−5.756	<.001

Note: Measurements that conform to the normal distribution were expressed as mean ± standard deviations (*x̄* ± s), measurements that do not meet the normal distribution were expressed as median (*P*_25_–*P*_75_). LDL-C = low-density lipoprotein cholesterol, TG = triglycerides, TC = total cholesterol.

### 3.2. Analysis of risk factors for carotid plaque in postmenopausal women

With carotid plaque as the dependent variable, binary logistic regression analysis after adjusting for confounders such as height, age, and waist-to-hip ratio showed that age, history of hypertension, and MHR were independent risk factors for the development of carotid plaque in postmenopausal women (odds ratio [OR] = 1.255, confidence interval [CI] 3.118–1.795, all *P* *<* .05), as shown in Table 2.

**Table 2:** Logistic regression analysis of factors influencing the development of carotid atherosclerosis in postmenopausal women (n = 214).

Projects	OR	95% CI	*P*-value	aOR value	95% CI	*P*-value
Age	1.04	1.03–1.04	<.001	1.255	1.16–1.36	<.001
With hypertension	0.63	0.55–0.72	<.001	3.118	1.19–8.18	.021
MHR	1.19	1.12–1.26	<.001	1.795	1.20–2.69	.005

aOR = adjusted odds ratio, CI = confidence interval, MHR = monocytes to high-density lipoprotein cholesterol ratio, OR = odds ratio.

### 3.3. Values of monocytes to high-density lipoprotein cholesterol ratio in screening for carotid plaque development in postmenopausal women

**Figure 1.**
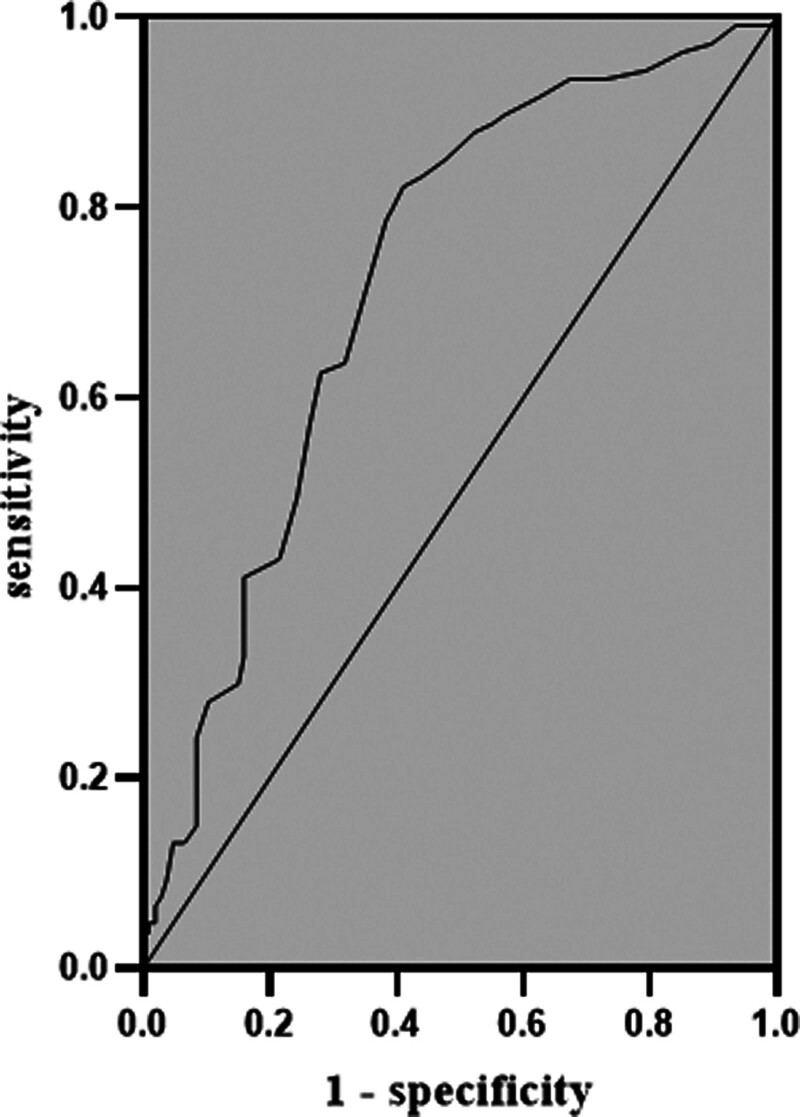
ROC curve for MHR predicting carotid atherosclerosis (n = 214). MHR = monocytes to high-density lipoprotein cholesterol ratio, ROC = receiver operating characteristics.

The area under the ROC curve for MHR predicting carotid plaque development in postmenopausal women was 0.724 (95% CI 0.656–0.793, *P* < .01), and the optimal cut-point value for MHR predicting carotid AS in postmenopausal women was 0.265, with a sensitivity of 82.2% and specificity of 58.9%, as shown in Table 3 and Figure 1.

**Table 3:** Diagnostic values of MHR for the development of carotid atherosclerosis in postmenopausal women.

Project	AUC	Sensitivity	Specificity	PPV	NPV	SE	Yoden Index	*P*-value	95% CI	Optimal cutoff value
MHR	0.724	0.822	0.589	0.827	0.580	0.035	0.411	<.001	0.66–0.79	0.265

AUC = area under the curve, CI = confidence interval, MHR = monocytes to high-density lipoprotein cholesterol ratio, NPV = negative predictive value, PPV = positive predictive value, SE = standard errors.

## 4. Discussion

Menopause, a time of great transition for women physically and mentally, is associated with an increase in risk for cardiovascular diseases due to the changes in the hormonal status, as well as the effects of aging and metabolic imbalances.^[1]^ Epidemiological studies have found that menopausal females are more susceptible to cardiovascular diseases than age-matched males.^[7]^ AS-associated morbidity and mortality are significantly prominent in postmenopausal women.^[8]^ In clinical practice, the underdiagnosis rate of cardiovascular diseases is high in postmenopausal female patients, with a low treatment rate. Therefore, the prevention of cardiovascular disease in older women should receive attention and focus.

Carotid plaque as a hallmark of carotid AS contributes to the prediction of atherosclerotic cardiovascular diseases and exhibits independent predictive value for cardiovascular diseases.^[9]^ As a systemic disease, AS inevitably detriments the large arteries in the circulation: the carotid and coronary arteries, leading to myocardial ischemia, cerebral insufficiency, and even critical illnesses such as myocardial infarction and stroke.^[10]^ Yoichi Inaba et al conducted a meta-analysis of 11 studies based on a population of 54,336 patients and reported that carotid plaque was more accurate in detecting coronary artery disease compared with carotid IMT.^[11]^

MHR has been identified as a novel marker that dynamically reflects the trend of inflammation and oxidative stress.^[12,13]^ MHR is determined by calculating the ratio of monocytes to HDL-C. Monocytes have pro-inflammatory phenotypes and infiltrates early atherosclerotic plaques, while HDL-C serves as a protective factor of AS by regulating monocyte activation, inhibiting the oxidation of LDL, defending endothelial cells from oxidative stress and inflammation.^[4]^ Our study revealed that the carotid plaque group had higher levels of pro-inflammatory factors such as leukocytes, neutrophils, lymphocytes, and monocytes but lower levels of antiinflammatory indicator HDL-C compared to the no carotid plaque group, and moreover, the carotid plaque group showed significantly higher MHR values, which were in line with the findings of Leening et al^[14]^ The possible reasons are as follows: on the one hand, estrogen has antiinflammatory effects, but the levels of estrogen in postmenopausal women decrease, resulting in a corresponding decrease in antiinflammatory capacity. On the other hand, various inflammatory factors release oxidative free radicals and pro-inflammatory mediators, causing vascular endothelial damage.^[15]^ Our binary logistic regression analysis after correcting for confounding factors unveiled that the single inflammatory factor of leukocyte lineage was not an independent risk factor for carotid AS in postmenopausal women, but age, history of hypertension, and MHR were independent risk factors for carotid plaque formation in postmenopausal women.

Age and hypertension have been shown to increase the risk of carotid plaque.^[16,17]^ A study conducted by Wang Wei et al in the general middle-aged and elderly population showed that the trend of carotid plaque formation with age was predominantly common in postmenopausal women and was directly related to the increased risk of AS.^[18]^ Consistently, the present study found that patients in the carotid plaque group were older than those in the no carotid plaque group. Also, this study identified hypertension as an independent risk factor for AS. Hypertension is a low-grade inflammatory disease. There exists a causal relationship between hypertension and inflammatory factors that trigger AS,^[19,20]^ and excessive inflammation leads to the formation, development and rupture of atherosclerotic plaques.

This study preliminarily explores the value of MHR in predicting carotid plaque development in postmenopausal women. According to the ROC curve, the optimal cut point value of MHR in predicting carotid AS in postmenopausal women was 0.265, with a sensitivity of 82.2%, a specificity of 58.9%, and an area under the curve of 0.724 (95% CI 0.656–0.793, *P* < .01). It is suggested that MHR ≥0.265 can better predict the occurrence of carotid plaque in postmenopausal women.

There are some limitations in this study. First of all, the cross-sectional design can only explain the correlation between MHR and AS in postmenopausal women. Secondly, this study is a single-center study with a small sample size, which does not rule out the existence of selection bias. Large-scale and multi-center studies are still needed to further supplement more clinical data to clarify the role of MHR.

## 5. Conclusion

In conclusion, MHR can independently contribute to the prediction of carotid plaque development in postmenopausal women. The MHR indicator is simple and easy to obtain, and its application in predicting carotid AS in postmenopausal women can be promoted at all levels of healthcare, including the community healthcare.

## Acknowledgments

We would like to acknowledge the reviewers for their helpful comments on this paper.

## Author contributions

**Conceptualization:** Jing Guo.

**Data curation:** Haibo Qin.

**Formal analysis:** Jing Guo, Xianxian Li.

**Methodology:** Jing Guo.

**Writing – original draft:** Jing Guo.

**Writing – review & editing:** Jing Guo.
